# Crystal structure and Hirshfeld surface analysis of 7-eth­oxy-5-methyl-2-(pyridin-3-yl)-11,12-di­hydro-5,11-methano­[1,2,4]triazolo[1,5-*c*][1,3,5]benzoxadiazo­cine

**DOI:** 10.1107/S2056989018002621

**Published:** 2018-02-20

**Authors:** Ercan Aydemir, Sevgi Kansiz, Mustafa Kemal Gumus, Nikolay Yu. Gorobets, Necmi Dege

**Affiliations:** a T.R. Ministry of Forestry and Water Affairs, 11th Regional Directorate, 55030, Ilkadım, Samsun, Turkey; b Artvin Coruh University, Science-Technology Research and Application Center, Artvin 08000, Turkey; c Ondokuz Mayıs University, Faculty of Arts and Sciences, Department of Physics, 55139, Kurupelit, Samsun, Turkey; d SSI "Institute for Single Crystals" of National Academy of Sciences of Ukraine, 60 Nauky Ave., Kharkiv 61072, Ukraine; e V.N. Karazin Kharkiv National University, Svobody sq. 4, Kharkiv 61077, Ukraine

**Keywords:** crystal structure, Biginelli condensation, benzoxa­diazo­cine, Hirshfeld surfaces

## Abstract

The title compound, C_19_H_19_N_5_O_2_, was prepared by the reaction of 3-amino-5-(pyridin-3-yl)-1,2,4-triazole with acetone and 2-hy­droxy-3-eth­oxy­benzaldehyde. It crystallizes from ethanol in a tetra­gonal space group, with one mol­ecule in the asymmetric unit. The 1,2,4-triazole five-membered ring is planar (maximum deviation = 0.0028 Å). The pyridine and phenyl rings are also planar with maximum deviations of 0.0091 and 0.0094 Å, respectively. In the crystal, N—H⋯N hydrogen bonds link the mol­ecules into supra­molecular chains propagating along the *c-*axis direction. Hirshfeld surface analysis and two-dimensional fingerprint plots have been used to analyse the inter­molecular inter­actions present in the crystal.

## Abstract 2

In the crystal, N—H⋯N hydrogen bonds link the mol­ecules into the supra­molecular chains propagating along the *c-*axis direction.

## Chemical context   

The title compound represents a conformationally restricted analogue of so-called Biginelli compounds known to exhibit multiple pharmacological activities. It was selected for a single-crystal X-ray analysis in order to probe the chemical and spatial requirements of some kinds of activity. 4-Aryl-3,4-di­hydro­pyrimidine-2(1*H*)-ones and -thio­nes, known as Bigin­elli compounds, display a wide spectrum of significant pharma­cological activities (Kappe, 2000 ▸). For example, these pyrimidine derivatives were assayed as anti­hypertensive agents, selective α_1a_-adrenergic receptor antagonists, neuropeptide Y antagonists and were used as a lead for the development of anti­cancer drugs (Kappe, 2000 ▸). The Biginelli products have also been found to be potent hepatitis B replication inhibitors (Deres *et al.*, 2003 ▸).

Recently, the ability of oxygen-bridged azolo­pyrimidine derivatives to inhibit Eg5 activity has been examined (Svetlík *et al.*, 2010 ▸). As each of the above activities originates from stereo-selective binding of the drug mol­ecule to its specific receptor, it is of inter­est to design a conformationally restricted probe mol­ecule in order to examine geometric requirements of the given receptor binding site.

Since we had previously synthesized such a rigid type of oxygen-bridged triazolo-pyrimidine derivative, (I) (Gümüş *et al.*, 2017 ▸), we decided to examine the structure of this heterocyclic system by X-ray analysis. A novel Biginelli-like assembly of 3-amino-5-(pyridin-3-yl)-1,2,4-triazole with acetone and 2-hy­droxy-3-eth­oxy­benzaldehyde has been developed to enable easy access to 7-eth­oxy-5-methyl-2-(pyri­din-3-yl)-11,12-di­hydro-5,11-methano­[1,2,4]triazolo[1,5-*c*][1,3,5]benzoxa­diazo­cine compounds as representatives of a new class of heterocycles.
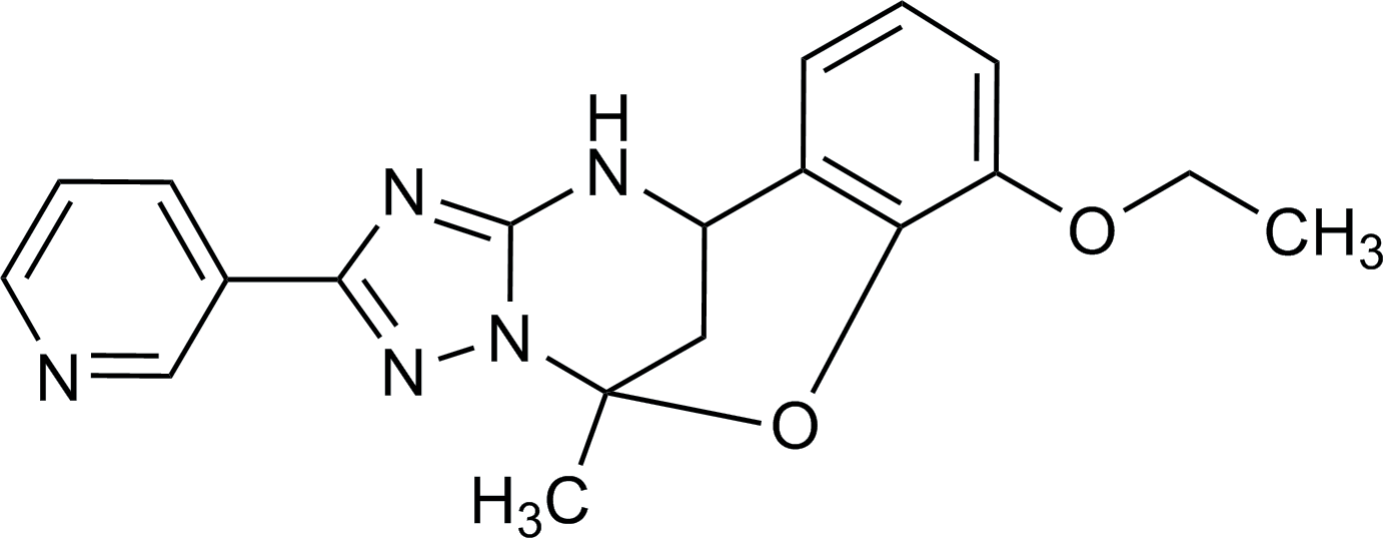


## Structural commentary   

**Figure 1:**
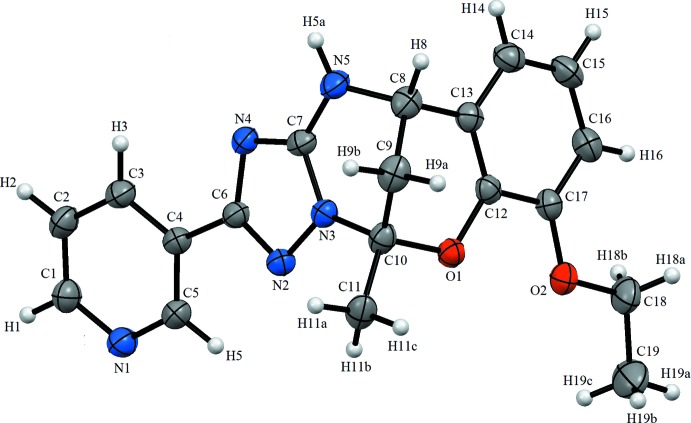
The mol­ecular structure of the title compound, showing the atom labelling. Displacement ellipsoids are drawn at the 30% probability level.

The asymmetric unit of the title compound contains one independent mol­ecule (Fig. 1 ▸). In the 1,2,4-triazole ring, the average C=N and C—N bond lengths are 1.324 and 1.355 Å, respectively, while the N—N bond is 1.389 (4) Å. These values consistent with literature values (Şen *et al.*, 2017*a* ▸,*b* ▸; Atalay *et al.*, 2004 ▸). The 1,2,4-triazole ring is planar with a maximum deviation of 0.0028 Å. The N1/C1–C5 and C12–C17 rings are also planar with maximum deviations of 0.0091 and 0.0094 Å, respectively. The dihedral angle between the N1/C1–C5 and C6/N2/N3/C7/N4 rings is 13.1 (2)°, while the latter ring is inclined to the N3/C10–C8/N5/C7 plane by 6.87 (15)°. The C12–C17 and N3/C10–C8/N5/C7 planes form dihedral angles of 7.8 (2) and 88.82 (12)°, respectively, with the C9/C10/O1/C12/C13 plane.

## Supra­molecular features   

**Figure 2:**
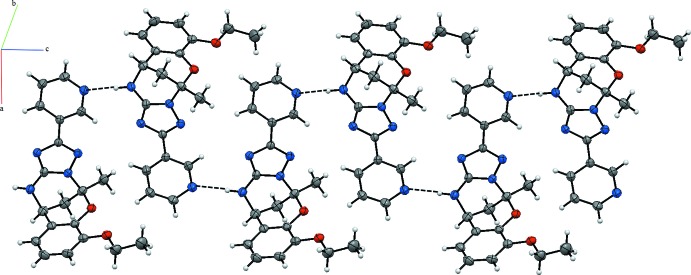
A partial view of the crystal packing of the title compound. Dashed lines denote the inter­molecular N—H⋯N hydrogen bonds.

In the crystal, the N—H⋯N hydrogen bonds link the mol­ecules, forming the supra­molecular chains propagating along the *c*-axis direction (Table 1 ▸, Fig. 2 ▸).

## Hirshfeld surface analysis   

**Figure 3:**
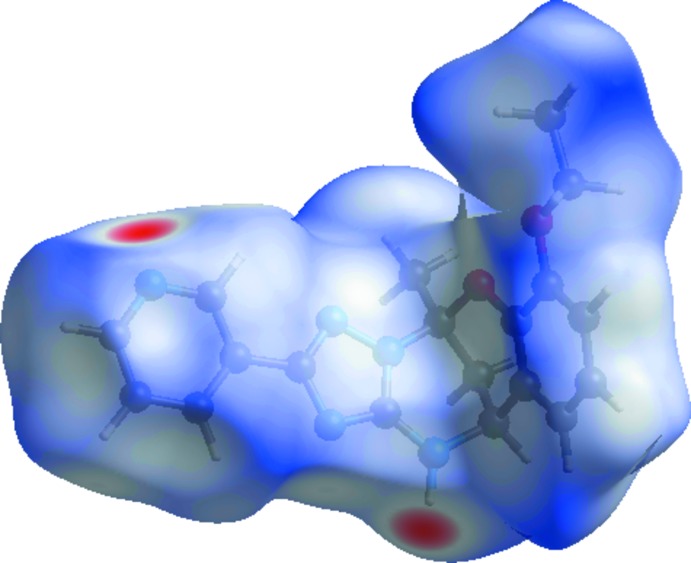
The Hirshfeld surface of C_19_H_19_N_5_O_2_ mapped with *d*_norm_.

*Crystal Explorer17.5* (Turner *et al.*, 2017 ▸) was used to analyse the inter­actions in the crystal; fingerprint plots mapped over *d*_norm_ (Figs. 3 ▸ and 4 ▸) were generated. The mol­ecular Hirshfeld surfaces were obtained using a standard (high) surface resolution with the three-dimentional *d*_norm_ surfaces mapped over a fixed colour scale of −0.484 (red) to 1.652 (blue). There are two red spots in the *d*_norm_ surface (Fig. 3 ▸), which are on the N-acceptor atoms involved in the inter­actions listed in Table 1 ▸. The red spots indicate the regions of donor–acceptor inter­actions (Kansiz *et al.*, 2018 ▸ *;* Şen *et al.*, 2017*a* ▸,*b* ▸, 2018 ▸; Yaman *et al.*, 2018 ▸).

**Figure 5:**
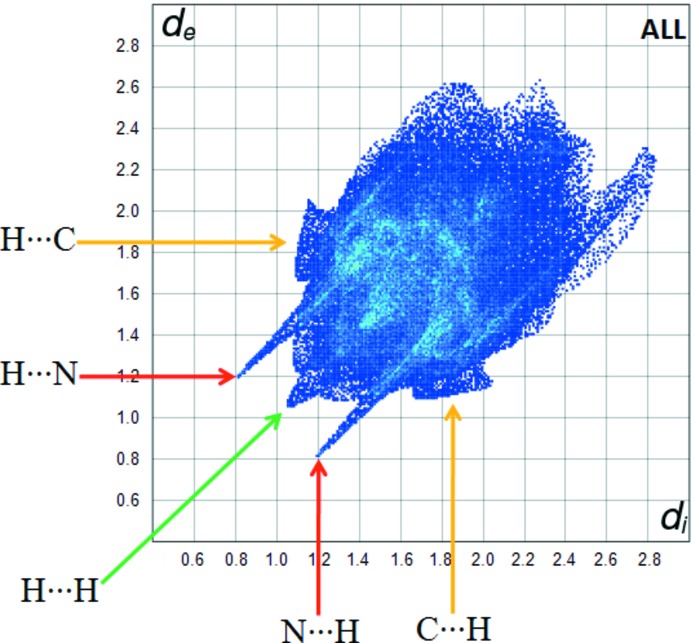
Fingerprint plot of the title compound.

**Figure 6:**
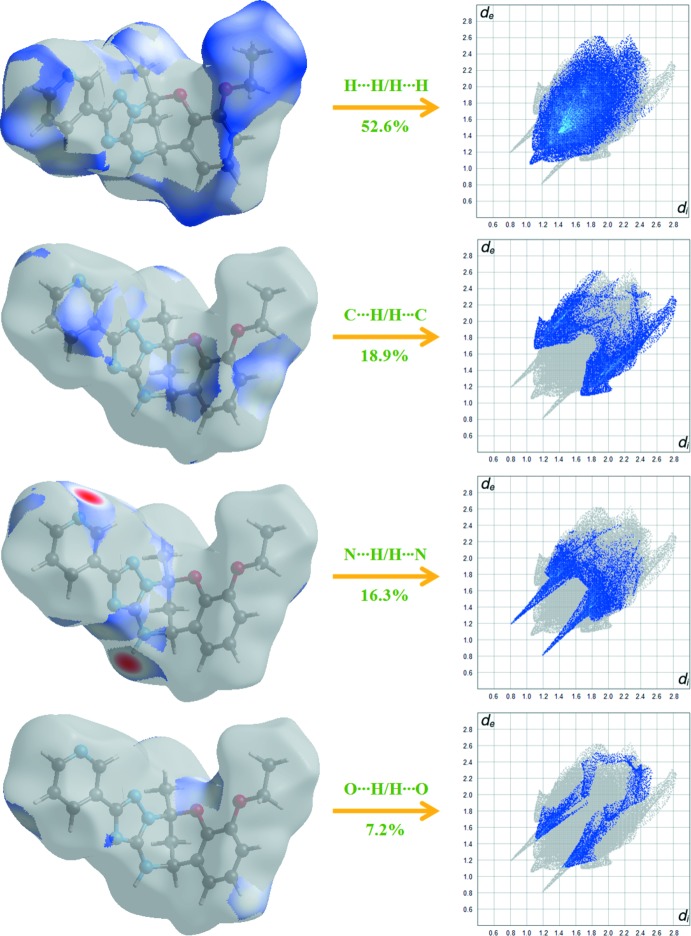
Two-dimensional fingerprint plots with a *d*_norm_ view of the H⋯H/H⋯H (52.6%), C⋯H/H⋯C (18.9%), N⋯H/H⋯N (16.3%) and O⋯H/H⋯O (7.2%) contacts in the title compound.

The inter­molecular inter­actions of the title compound are shown in the 2D fingerprint plots shown in Fig. 5 ▸. The H⋯H inter­actions appear in the middle of the scattered points in the two-dimensional fingerprint plots with a contribution to the overall Hirshfeld surface of 52.6% (Fig. 6 ▸). The contribution from the N⋯H/H⋯N contacts, corresponding to the N—H⋯N inter­action, is represented by a pair of sharp spikes characteristic of a strong hydrogen-bond inter­action (16.3%). The whole fingerprint region and all other inter­actions, which are a combination of *d*_e_ and *d*_i_, are displayed in Fig. 6 ▸.

## Database survey   

There are no direct precedents for the structure of (I) in the crystallographic literature (CSD Version 5.38; Groom *et al.*, 2016 ▸). However, there are several precedents for the triazolobenzoxa­diazo­cines, including the structures of 5-(2-hy­droxy­phen­yl)-7-methyl-4,5,6,7-tetra­hydro­[1,2,4]triazolo[1,5-*a*]pyrimidin-7-ol (Gorobets *et al.*, 2010 ▸), ethyl 7-chloro­methyl-5-(2-chloro­phen­yl)-7-hy­droxy-2-methyl­sulfanyl-4,5,6,7-tetra­hydro-1,2,4-triazolo[1,5-*a*]pyrimidine-6-carboxyl­ate (Huang, 2009 ▸) and methyl 5′-(2-hy­droxy­phen­yl)-5′,6′-di­hydro-4′*H*-spiro­[chromene-2,7′-[1,2,4]triazolo[1,5-*a*]pyrimidine]-3-carboxyl­ate (Kettmann & Světlík, 2011 ▸).

## Synthesis and crystallization   

**Figure 7:**
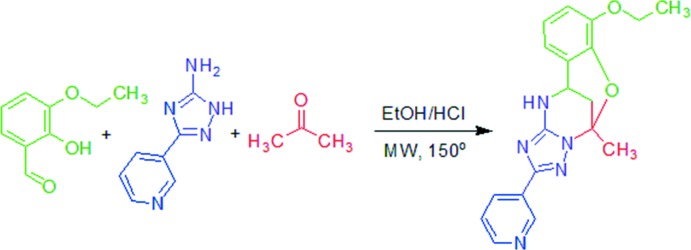
Synthesis of the title compound.

The synthesis of the title compound (Fig. 7 ▸) was described by Gümüş *et al.* (2017 ▸). 3-Amino-5-(pyridin-3-yl)-1,2,4-triazole(1.0 mmol), 2-hy­droxy-3-eth­oxy­benzaldehyde (1.0 mmol), acetone (0.22 mL, 3.0 mmol), and abs. EtOH (2.0 mL) were mixed in a microwave process vial, and then a 4 *N* solution of HCl in dioxane (0.07 mL, 0.3 mmol) was added. The mixture was irradiated at 423 K for 30 min. The reaction mixture was cooled by an air flow and stirred for 24 h at room temperature for complete precipitation of the product. The precipitate was filtered off, washed with EtOH (1.0 mL) and Et_2_O (3 × 1.0 mL), and dried. Compound (I) was obtained in the form of a white solid. It was recrystallized from ethanol.

## Refinement   

Crystal data, data collection and structure refinement details are summarized in Table 2 ▸. H atoms were positioned geometrically [N—H = 0.86 Å, C—H = 0.93 (aromatic), 0.96 (meth­yl) and 0.97 (methyl­ene) Å] and refined using a riding model, with *U*_iso_(H) = 1.2*U*_eq_(N, C) and 1.5*U*_eq_(methyl C).

## Supplementary Material

Crystal structure: contains datablock(s) I, global. DOI: 10.1107/S2056989018002621/xu5917sup1.cif

Structure factors: contains datablock(s) I. DOI: 10.1107/S2056989018002621/xu5917Isup2.hkl

Supporting information file. DOI: 10.1107/S2056989018002621/xu5917Isup3.cml

CCDC reference: 1820439

Additional supporting information:  crystallographic information; 3D view; checkCIF report

## Figures and Tables

**Figure 4:**
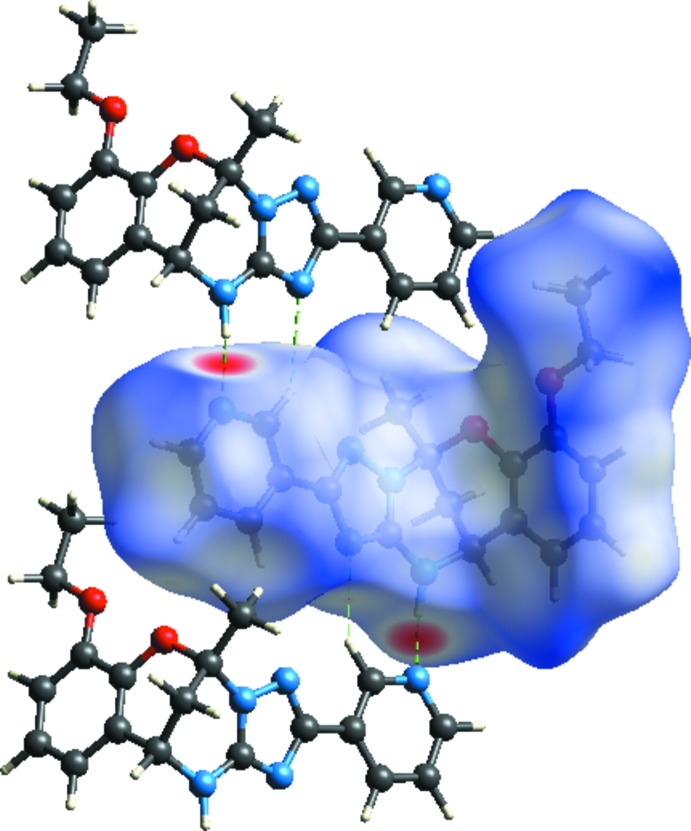
*d*_norm_ mapped on Hirshfeld surfaces to visualize the inter­molecular inter­actions of C_19_H_19_N_5_O_2_.

**Table 1:** Hydrogen-bond geometry (Å, °)

*D*—H⋯*A*	*D*—H	H⋯*A*	*D*⋯*A*	*D*—H⋯*A*
N5—H5*A*⋯N1^i^	0.86	2.13	2.907 (4)	149

Symmetry code: (i) 
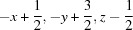
.

**Table 2:** Experimental details

Crystal data	
Chemical formula	C_19_H_19_N_5_O_2_
*M*_r_	349.39
Crystal system, space group	Tetragonal, *I* 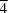
Temperature (K)	293
*a*, *c* (Å)	17.1509 (8), 11.9033 (7)
*V* (Å^3^)	3501.4 (4)
*Z*	8
Radiation type	Mo *K*α
μ (mm^−1^)	0.09
Crystal size (mm)	0.54 × 0.34 × 0.16

Data collection	
Diffractometer	Stoe IPDS 2
Absorption correction	Integration (*X-RED32*; Stoe & Cie, 2002 ▸)
*T*_min_, *T*_max_	0.959, 0.984
No. of measured, independent and observed [*I* > 2σ(*I*)] reflections	8018, 3629, 2449
*R*_int_	0.053
(sin θ/λ)_max_ (Å^−1^)	0.628

Refinement	
*R*[*F* ^2^ > 2σ(*F* ^2^)], *wR*(*F* ^2^), *S*	0.042, 0.088, 0.90
No. of reflections	3629
No. of parameters	236
H-atom treatment	H-atom parameters constrained
Δρ_max_, Δρ_min_ (e Å^−3^)	0.15, −0.12
Absolute structure	Refined as an inversion twin.
Absolute structure parameter	−3 (2)

Computer programs: *X-AREA* and *X-RED* (Stoe & Cie, 2002 ▸), *SHELXL2017* (Sheldrick, 2008 ▸), *ORTEP-3 for Windows* and *WinGX* (Farrugia, 2012 ▸) and *PLATON* (Spek, 2009 ▸).

## supplementary crystallographic information

### Crystal data

**Table:**

C_19_H_19_N_5_O_2_	*D*_x_ = 1.326 Mg m^−^^3^
*M**_r_* = 349.39	Mo *K*α radiation, λ = 0.71073 Å
Tetragonal, *I*4	Cell parameters from 8727 reflections
*a* = 17.1509 (8) Å	θ = 1.7–27.6°
*c* = 11.9033 (7) Å	µ = 0.09 mm^−^^1^
*V* = 3501.4 (4) Å^3^	*T* = 293 K
*Z* = 8	Prism, colorless
*F*(000) = 1472	0.54 × 0.34 × 0.16 mm

### Data collection

**Table:**

Stoe IPDS 2 diffractometer	3629 independent reflections
Radiation source: sealed X-ray tube, 12 x 0.4 mm long-fine focus	2449 reflections with *I* > 2σ(*I*)
Detector resolution: 6.67 pixels mm^-1^	*R*_int_ = 0.053
rotation method scans	θ_max_ = 26.5°, θ_min_ = 1.7°
Absorption correction: integration (X-RED32; Stoe & Cie, 2002)	*h* = −20→21
*T*_min_ = 0.959, *T*_max_ = 0.984	*k* = −21→21
8018 measured reflections	*l* = −14→13

### Refinement

**Table:**

Refinement on *F*^2^	Hydrogen site location: inferred from neighbouring sites
Least-squares matrix: full	H-atom parameters constrained
*R*[*F*^2^ > 2σ(*F*^2^)] = 0.042	*w* = 1/[σ^2^(*F*_o_^2^) + (0.0372*P*)^2^] where *P* = (*F*_o_^2^ + 2*F*_c_^2^)/3
*wR*(*F*^2^) = 0.088	(Δ/σ)_max_ < 0.001
*S* = 0.90	Δρ_max_ = 0.15 e Å^−^^3^
3629 reflections	Δρ_min_ = −0.12 e Å^−^^3^
236 parameters	Absolute structure: Refined as an inversion twin.
0 restraints	Absolute structure parameter: −3 (2)

### Special details

**Table:**

Geometry. All esds (except the esd in the dihedral angle between two l.s. planes) are estimated using the full covariance matrix. The cell esds are taken into account individually in the estimation of esds in distances, angles and torsion angles; correlations between esds in cell parameters are only used when they are defined by crystal symmetry. An approximate (isotropic) treatment of cell esds is used for estimating esds involving l.s. planes.
Refinement. Refined as a 2-component inversion twin.

### Fractional atomic coordinates and isotropic or equivalent isotropic displacement parameters (Å^2^)

**Table:**

	*x*	*y*	*z*	*U*_iso_*/*U*_eq_	
C1	0.0390 (2)	0.7259 (2)	0.4925 (3)	0.0581 (10)	
H1	−0.014021	0.716332	0.501878	0.070*	
C2	0.0662 (2)	0.7372 (2)	0.3863 (3)	0.0597 (10)	
H2	0.032912	0.733641	0.324888	0.072*	
C3	0.1444 (2)	0.7541 (2)	0.3716 (3)	0.0558 (9)	
H3	0.164641	0.761427	0.299904	0.067*	
C4	0.19186 (19)	0.76002 (19)	0.4641 (3)	0.0451 (8)	
C5	0.1594 (2)	0.7450 (2)	0.5683 (3)	0.0524 (9)	
H5	0.191757	0.746889	0.630959	0.063*	
C6	0.27446 (19)	0.78337 (18)	0.4538 (3)	0.0433 (8)	
C7	0.3820 (2)	0.81015 (19)	0.3807 (3)	0.0452 (8)	
C8	0.5188 (2)	0.8326 (2)	0.3714 (3)	0.0530 (9)	
H8	0.557656	0.853590	0.319160	0.064*	
C9	0.5062 (2)	0.8898 (2)	0.4669 (4)	0.0575 (10)	
H9A	0.555879	0.903724	0.500282	0.069*	
H9B	0.481787	0.936913	0.438785	0.069*	
C10	0.4545 (2)	0.85180 (19)	0.5536 (3)	0.0484 (8)	
C11	0.4295 (2)	0.9041 (2)	0.6485 (4)	0.0620 (11)	
H11A	0.401451	0.947929	0.618728	0.093*	
H11B	0.396382	0.875548	0.698823	0.093*	
H11C	0.474656	0.922184	0.688324	0.093*	
C12	0.53691 (18)	0.74016 (18)	0.5322 (3)	0.0432 (8)	
C13	0.54834 (18)	0.75667 (19)	0.4207 (3)	0.0460 (8)	
C14	0.5883 (2)	0.7029 (2)	0.3536 (3)	0.0557 (10)	
H14	0.596039	0.713003	0.277630	0.067*	
C15	0.6162 (2)	0.6352 (2)	0.4004 (4)	0.0631 (11)	
H15	0.642215	0.599352	0.355389	0.076*	
C16	0.6062 (2)	0.6195 (2)	0.5133 (3)	0.0576 (10)	
H16	0.625672	0.573495	0.543568	0.069*	
C17	0.56737 (19)	0.6719 (2)	0.5813 (3)	0.0471 (8)	
C18	0.5850 (3)	0.5943 (3)	0.7453 (4)	0.0716 (12)	
H18A	0.641332	0.593704	0.738080	0.086*	
H18B	0.564269	0.547848	0.709738	0.086*	
C19	0.5622 (3)	0.5965 (3)	0.8665 (4)	0.1006 (17)	
H19A	0.582492	0.551306	0.903941	0.151*	
H19B	0.583133	0.642681	0.900739	0.151*	
H19C	0.506407	0.597026	0.872521	0.151*	
N1	0.08383 (17)	0.72774 (19)	0.5838 (3)	0.0572 (8)	
N2	0.31602 (16)	0.80437 (15)	0.5418 (2)	0.0458 (7)	
N3	0.38723 (15)	0.82148 (16)	0.4920 (2)	0.0442 (7)	
N4	0.31108 (15)	0.78598 (17)	0.3519 (2)	0.0467 (7)	
N5	0.44380 (16)	0.82375 (18)	0.3130 (2)	0.0542 (8)	
H5A	0.439554	0.826988	0.241203	0.065*	
O1	0.49588 (12)	0.78802 (13)	0.60565 (18)	0.0468 (6)	
O2	0.55363 (14)	0.66252 (14)	0.6933 (2)	0.0567 (7)	

### Atomic displacement parameters (Å^2^)

**Table:**

	*U*^11^	*U*^22^	*U*^33^	*U*^12^	*U*^13^	*U*^23^
C1	0.045 (2)	0.071 (3)	0.058 (3)	−0.0032 (17)	−0.0068 (19)	−0.002 (2)
C2	0.055 (2)	0.077 (3)	0.048 (2)	−0.0014 (19)	−0.0144 (18)	−0.001 (2)
C3	0.055 (2)	0.070 (2)	0.042 (2)	0.0003 (18)	−0.0024 (18)	0.0043 (18)
C4	0.0452 (19)	0.051 (2)	0.0389 (19)	0.0027 (15)	−0.0019 (16)	−0.0025 (16)
C5	0.050 (2)	0.065 (2)	0.043 (2)	0.0048 (17)	−0.0047 (17)	−0.0016 (18)
C6	0.0474 (19)	0.0454 (19)	0.0370 (19)	0.0039 (14)	−0.0035 (16)	−0.0014 (16)
C7	0.052 (2)	0.046 (2)	0.037 (2)	0.0000 (16)	−0.0011 (17)	0.0026 (16)
C8	0.050 (2)	0.054 (2)	0.055 (2)	−0.0108 (17)	−0.0034 (18)	0.0068 (19)
C9	0.062 (2)	0.0416 (19)	0.069 (3)	−0.0079 (16)	−0.012 (2)	0.0020 (19)
C10	0.052 (2)	0.0433 (18)	0.050 (2)	0.0020 (15)	−0.0100 (17)	−0.0025 (17)
C11	0.070 (3)	0.051 (2)	0.064 (3)	0.0125 (18)	−0.020 (2)	−0.0181 (19)
C12	0.0350 (17)	0.0465 (19)	0.048 (2)	−0.0012 (14)	−0.0029 (15)	−0.0062 (17)
C13	0.0388 (19)	0.050 (2)	0.049 (2)	−0.0059 (15)	−0.0008 (16)	−0.0023 (17)
C14	0.048 (2)	0.066 (2)	0.054 (2)	−0.0040 (18)	0.0081 (18)	−0.0043 (19)
C15	0.056 (2)	0.065 (3)	0.068 (3)	0.0102 (19)	0.014 (2)	−0.013 (2)
C16	0.048 (2)	0.056 (2)	0.069 (3)	0.0077 (17)	0.0038 (19)	−0.001 (2)
C17	0.0395 (19)	0.048 (2)	0.053 (2)	0.0002 (15)	−0.0013 (16)	0.0001 (17)
C18	0.070 (3)	0.072 (3)	0.073 (3)	0.020 (2)	−0.002 (2)	0.020 (2)
C19	0.101 (4)	0.119 (4)	0.082 (3)	0.032 (3)	0.009 (3)	0.039 (3)
N1	0.0495 (18)	0.075 (2)	0.0473 (19)	−0.0042 (15)	−0.0009 (15)	0.0003 (16)
N2	0.0471 (17)	0.0498 (16)	0.0404 (17)	0.0034 (12)	−0.0027 (14)	−0.0044 (13)
N3	0.0414 (16)	0.0491 (16)	0.0422 (18)	−0.0011 (12)	−0.0038 (13)	−0.0030 (13)
N4	0.0436 (17)	0.0571 (18)	0.0393 (17)	0.0005 (14)	−0.0050 (13)	0.0033 (13)
N5	0.0486 (18)	0.073 (2)	0.0410 (16)	−0.0050 (15)	−0.0034 (14)	0.0104 (16)
O1	0.0494 (13)	0.0459 (13)	0.0451 (13)	0.0082 (10)	−0.0054 (11)	−0.0044 (11)
O2	0.0557 (15)	0.0585 (16)	0.0559 (16)	0.0140 (12)	−0.0017 (13)	0.0087 (13)

### Geometric parameters (Å, º)

**Table:**

C1—N1	1.331 (5)	C10—C11	1.504 (5)
C1—C2	1.362 (5)	C11—H11A	0.9600
C1—H1	0.9300	C11—H11B	0.9600
C2—C3	1.384 (5)	C11—H11C	0.9600
C2—H2	0.9300	C12—C13	1.372 (5)
C3—C4	1.373 (5)	C12—O1	1.390 (4)
C3—H3	0.9300	C12—C17	1.409 (5)
C4—C5	1.383 (5)	C13—C14	1.399 (5)
C4—C6	1.477 (4)	C14—C15	1.374 (5)
C5—N1	1.343 (4)	C14—H14	0.9300
C5—H5	0.9300	C15—C16	1.380 (6)
C6—N2	1.317 (4)	C15—H15	0.9300
C6—N4	1.367 (4)	C16—C17	1.380 (5)
C7—N4	1.330 (4)	C16—H16	0.9300
C7—N3	1.342 (4)	C17—O2	1.364 (4)
C7—N5	1.352 (4)	C18—O2	1.428 (4)
C8—N5	1.469 (4)	C18—C19	1.495 (6)
C8—C13	1.515 (5)	C18—H18A	0.9700
C8—C9	1.517 (5)	C18—H18B	0.9700
C8—H8	0.9800	C19—H19A	0.9600
C9—C10	1.509 (5)	C19—H19B	0.9600
C9—H9A	0.9700	C19—H19C	0.9600
C9—H9B	0.9700	N2—N3	1.389 (4)
C10—O1	1.443 (4)	N5—H5A	0.8600
C10—N3	1.463 (4)		
			
N1—C1—C2	123.7 (3)	H11A—C11—H11C	109.5
N1—C1—H1	118.1	H11B—C11—H11C	109.5
C2—C1—H1	118.1	C13—C12—O1	124.0 (3)
C1—C2—C3	118.7 (4)	C13—C12—C17	121.3 (3)
C1—C2—H2	120.7	O1—C12—C17	114.7 (3)
C3—C2—H2	120.7	C12—C13—C14	119.1 (3)
C4—C3—C2	119.2 (3)	C12—C13—C8	120.3 (3)
C4—C3—H3	120.4	C14—C13—C8	120.6 (3)
C2—C3—H3	120.4	C15—C14—C13	119.7 (4)
C3—C4—C5	117.8 (3)	C15—C14—H14	120.1
C3—C4—C6	121.4 (3)	C13—C14—H14	120.1
C5—C4—C6	120.7 (3)	C14—C15—C16	121.1 (4)
N1—C5—C4	123.5 (3)	C14—C15—H15	119.5
N1—C5—H5	118.2	C16—C15—H15	119.5
C4—C5—H5	118.2	C17—C16—C15	120.3 (4)
N2—C6—N4	116.6 (3)	C17—C16—H16	119.9
N2—C6—C4	121.8 (3)	C15—C16—H16	119.9
N4—C6—C4	121.5 (3)	O2—C17—C16	125.5 (3)
N4—C7—N3	111.2 (3)	O2—C17—C12	116.0 (3)
N4—C7—N5	128.1 (3)	C16—C17—C12	118.5 (3)
N3—C7—N5	120.7 (3)	O2—C18—C19	107.4 (4)
N5—C8—C13	112.8 (3)	O2—C18—H18A	110.2
N5—C8—C9	107.2 (3)	C19—C18—H18A	110.2
C13—C8—C9	108.2 (3)	O2—C18—H18B	110.2
N5—C8—H8	109.5	C19—C18—H18B	110.2
C13—C8—H8	109.5	H18A—C18—H18B	108.5
C9—C8—H8	109.5	C18—C19—H19A	109.5
C10—C9—C8	108.5 (3)	C18—C19—H19B	109.5
C10—C9—H9A	110.0	H19A—C19—H19B	109.5
C8—C9—H9A	110.0	C18—C19—H19C	109.5
C10—C9—H9B	110.0	H19A—C19—H19C	109.5
C8—C9—H9B	110.0	H19B—C19—H19C	109.5
H9A—C9—H9B	108.4	C1—N1—C5	116.8 (3)
O1—C10—N3	109.5 (3)	C6—N2—N3	101.2 (3)
O1—C10—C11	105.7 (3)	C7—N3—N2	109.4 (3)
N3—C10—C11	111.3 (3)	C7—N3—C10	126.8 (3)
O1—C10—C9	109.4 (3)	N2—N3—C10	123.7 (3)
N3—C10—C9	105.9 (3)	C7—N4—C6	101.6 (3)
C11—C10—C9	115.1 (3)	C7—N5—C8	115.0 (3)
C10—C11—H11A	109.5	C7—N5—H5A	122.5
C10—C11—H11B	109.5	C8—N5—H5A	122.5
H11A—C11—H11B	109.5	C12—O1—C10	115.3 (2)
C10—C11—H11C	109.5	C17—O2—C18	117.1 (3)
			
N1—C1—C2—C3	−2.2 (6)	C2—C1—N1—C5	2.8 (6)
C1—C2—C3—C4	−0.9 (6)	C4—C5—N1—C1	−0.4 (5)
C2—C3—C4—C5	3.0 (5)	N4—C6—N2—N3	−0.8 (3)
C2—C3—C4—C6	−175.7 (3)	C4—C6—N2—N3	−179.3 (3)
C3—C4—C5—N1	−2.5 (5)	N4—C7—N3—N2	−0.2 (4)
C6—C4—C5—N1	176.2 (3)	N5—C7—N3—N2	179.3 (3)
C3—C4—C6—N2	166.1 (3)	N4—C7—N3—C10	−176.5 (3)
C5—C4—C6—N2	−12.5 (5)	N5—C7—N3—C10	3.0 (5)
C3—C4—C6—N4	−12.4 (5)	C6—N2—N3—C7	0.5 (3)
C5—C4—C6—N4	169.0 (3)	C6—N2—N3—C10	177.0 (3)
N5—C8—C9—C10	68.7 (3)	O1—C10—N3—C7	−101.2 (4)
C13—C8—C9—C10	−53.3 (4)	C11—C10—N3—C7	142.3 (4)
C8—C9—C10—O1	67.4 (3)	C9—C10—N3—C7	16.6 (4)
C8—C9—C10—N3	−50.5 (3)	O1—C10—N3—N2	83.0 (4)
C8—C9—C10—C11	−173.8 (3)	C11—C10—N3—N2	−33.5 (4)
O1—C12—C13—C14	−177.8 (3)	C9—C10—N3—N2	−159.2 (3)
C17—C12—C13—C14	2.6 (5)	N3—C7—N4—C6	−0.2 (4)
O1—C12—C13—C8	2.9 (5)	N5—C7—N4—C6	−179.7 (3)
C17—C12—C13—C8	−176.7 (3)	N2—C6—N4—C7	0.7 (4)
N5—C8—C13—C12	−98.3 (4)	C4—C6—N4—C7	179.2 (3)
C9—C8—C13—C12	20.1 (4)	N4—C7—N5—C8	−166.6 (3)
N5—C8—C13—C14	82.4 (4)	N3—C7—N5—C8	14.0 (5)
C9—C8—C13—C14	−159.2 (3)	C13—C8—N5—C7	70.4 (4)
C12—C13—C14—C15	−0.7 (5)	C9—C8—N5—C7	−48.7 (4)
C8—C13—C14—C15	178.6 (3)	C13—C12—O1—C10	9.4 (4)
C13—C14—C15—C16	−0.7 (6)	C17—C12—O1—C10	−171.0 (3)
C14—C15—C16—C17	0.3 (6)	N3—C10—O1—C12	71.7 (3)
C15—C16—C17—O2	179.6 (4)	C11—C10—O1—C12	−168.4 (3)
C15—C16—C17—C12	1.5 (5)	C9—C10—O1—C12	−43.9 (4)
C13—C12—C17—O2	178.7 (3)	C16—C17—O2—C18	2.8 (5)
O1—C12—C17—O2	−0.9 (4)	C12—C17—O2—C18	−179.0 (3)
C13—C12—C17—C16	−3.0 (5)	C19—C18—O2—C17	−179.6 (4)
O1—C12—C17—C16	177.4 (3)		

### Hydrogen-bond geometry (Å, º)

**Table:**

*D*—H···*A*	*D*—H	H···*A*	*D*···*A*	*D*—H···*A*
N5—H5*A*···N1^i^	0.86	2.13	2.907 (4)	149

Symmetry code: (i) −*x*+1/2, −*y*+3/2, *z*−1/2.
